# A model for sustainable, partnership-based telehealth services in rural India: An early process evaluation from Tuver village, Gujarat

**DOI:** 10.1371/journal.pone.0261907

**Published:** 2022-01-13

**Authors:** Shoba Ramanadhan, Krishnan Ganapathy, Lovakanth Nukala, Subramaniya Rajagopalan, John C. Camillus

**Affiliations:** 1 Department of Social and Behavioral Sciences, Harvard T.H. Chan School of Public Health, Boston, MA, United States of America; 2 Apollo Telemedicine Networking Foundation, Chennai, Tamil Nadu, India; 3 Safe World Rural Services, Chennai, Tamil Nadu, India; 4 Joseph M. Katz Graduate School of Business, University of Pittsburgh, Pittsburgh, PA, United States of America; Universidad Nacional Autonoma de Nicaragua Leon, NICARAGUA

## Abstract

**Background:**

Telehealth can improve access to high-quality healthcare for rural populations in India. However, rural communities often have other needs, such as sanitation or employment, to benefit fully from telehealth offerings, highlighting a need for systems-level solutions. A Business of Humanity approach argues that innovative solutions to wicked problems like these require strategic decision-making that attends to a) humaneness, e.g., equity and safety and b) humankind, or the needs and potential of large and growing markets comprised of marginalized and low-income individuals. The approach is expected to improve economic performance and long-term value creation for partners, thus supporting sustainability.

**Methods:**

A demonstration project was conducted in Tuver, a rural and tribal village in Gujarat, India. The project included seven components: a partnership that emphasized power-sharing and complementary contributions; telehealth services; health promotion; digital services; power infrastructure; water and sanitation; and agribusiness. Core partners included the academic partner, local village leadership, a local development foundation, a telehealth provider, and a design-build contractor. This early process evaluation relies on administrative data, field notes, and project documentation and was analyzed using a case study approach.

**Results:**

Findings highlight the importance of taking a systems perspective and engaging inter-sectoral partners through alignment of values and goals. Additionally, the creation of a synergistic, health-promoting ecosystem offers potential to support telehealth services in the long-term. At the same time, engaging rural, tribal communities in the use of technological advances posed a challenge, though local staff and intermediaries were effective in bridging disconnects.

**Conclusion:**

Overall, this early process evaluation highlights the promise and challenges of using a Business of Humanity approach for coordinated, sustainable community-level action to improve the health and well-being of marginalized communities.

## Introduction

Telehealth offers the opportunity to improve the health and well-being of marginalized and excluded communities, with benefits including cost savings and cost-effectiveness for patients/families and systems, health service access and utilization, health education, patient satisfaction, and quality of life [1,2]. Improving access is critical in India, where 59% of health workers and 80% of specialists practice in urban areas, which account for only 28% of the population [3]. Additionally, the private sector provides the bulk of care (78% of health expenditure) and patients typically pay out-of-pocket (71% of total health spending). As a result, low-income households experience significant barriers to care, often leading to catastrophic expenditures and unresolvable debt [4,5]. Telehealth offers an alternative by using information and communications technology to deliver services over a distance, as highlighted in the Ministry of Health and Family Welfare’s 5-year plans as a means to increase access to primary and specialist care for the country’s underserved populations [6–8].

Despite its potential, several barriers impede the implementation and sustainment of telehealth services. Substantial challenges exist at the patient level (e.g., computer literacy); staff/provider level (e.g., cultural and linguistic mismatch with patients); organization level (e.g., concerns about costs and reimbursement); and system level (e.g., limited data demonstrating rationale for investment) [2,9]. To benefit from telehealth services, patients and community members must also have access to public health services, including maternal and child health offerings, clean water, sanitation and hygiene, and nutrition, which are insufficient in many rural regions across India [10]. Together, these requirements highlight the need to take a long-term, systems-focused approach to design, deliver, and sustain telehealth initiatives in rural India.

One opportunity to support telehealth through systems change comes from cross-sectoral partnerships. These partnerships are expected to have positive impacts, as they bring to bear synergistic capacities, services, and resources of diverse actors to address issues of interest. Yet, unsuccessful partnerships can have wide-ranging negative consequences ranging from losing goodwill and reputation to wasting limited resources [11,12]. Successful cross-sectoral collaborations in India have improved service delivery for diverse health issues, including tuberculosis, HIV, and cancer prevention and treatment [13]. However, policymakers and leaders lack clarity regarding the best ways to design, implement, and sustain cross-sectoral collaborations for health [14]. Critical gaps in the literature relate to selecting members for a partnership, identifying meaningful goals for diverse partners, finding the best ways to manage partnerships, and examining the link between partnership models and outcomes of effectiveness, efficiency, and equity [15]. These challenges become all the more important when attempting to address the needs of a marginalized community.

To explore solutions, we drew on the Business of Humanity (BoH) approach, which proposes that strategic decision-making that explicitly includes criteria related to “humanity” will improve economic performance and the ability to create value over the long term. In this approach, humanity includes 1) humaneness, or focusing on safety, quality, sustainability, equity, and integrity and 2) humankind, or focusing on the needs and potential of large and growing markets among marginalized and socioeconomically disadvantaged groups. The strategy requires a commitment to a “moonshot” goal that addresses the needs of marginalized communities; proposes an innovative business model; stimulates and sustains innovation; creates alliances and partnerships with community, governmental, nonprofit, and academic institutions; and seeks to generate economic value across the entire value chain [16]. The goal is to shift the focus away from proximal challenges toward opportunities to influence the broader system to promote health and well-being over the long term. Using a demonstration project as an example, we are exploring the following questions: How can a BoH approach support the creation of a sustainable, cross-sector initiative centered on telehealth in rural India? What barriers and challenges limit this approach’s impact? This early process evaluation offers insight into applying this model and opportunities to adapt the initiative to increase its sustainability.

## Methods

### Project overview and setting

The University of Pittsburgh BoH Project, in association with Apollo Telemedicine Networking Foundation (ATNF), Safe World Rural Services (SRS), and Narottam Lalbhai Rural Development Fund (NLRDF) initiated the Tuver Health & Wellness Centre Project, referred to as the Tuver Project hereafter. The initiative seeks to demonstrate the feasibility of sustainably improving the quality of life in a marginalized community by systemically addressing the community’s basic needs—emphasizing health and wellness—and implementing a business model that provides income to the community plus a reasonable return to investors. The University of Pittsburgh team initiated the partnership, building on an existing relationship with NLRDF, which identified Tuver within its catchment area as a promising location for the project. NLRDF engaged village leadership to consider and commit to the partnership. Initial conversations began in 2016 and services launched in February 2019.

Tuver village is located in a tribal area; census data from 2011 described a medium-sized rural village, with about 1,000 residents, all of whom were members of a scheduled tribe, and roughly 48% of whom were literate [17]. As context, tribal communities in India have been marginalized and excluded from development opportunities, which is reflected in their higher rates of poverty, lower scores on the human development index, and insufficient access to high-quality healthcare services compared to the rest of the Indian population. Although there is tremendous diversity across the 700-plus tribal communities in India, these patterns offer important context [18–20]. Before the Tuver Project’s implementation, the tribal and rural communities in Tuver and adjacent villages obtained healthcare services primarily through local traditional healers and private clinics. The nearest primary health center was located in Unchidhanal, a journey of about 5 kms over mostly unpaved, rural roads. A community health center, private clinics, laboratory centers, and pharmacies were located in Khedbrahma, a major town 16 kilometers away, but specialists were limited to Gynecology, Pediatrics, Orthopedics, and General Medicine. Rural and tribal community members from Tuver visited either Himatnagar, 68 kms away, or Ahmedabad, 153 kms away, for all other medical specialties. The Indian government launched the Ayushman Bharat program–a national program to improve access to health and wellness services among underserved individuals–in 2018. The program offers health insurance to families and includes creating health and wellness centers across the country to provide primary care services [21,22]. However, service accessibility remained a challenge in Tuver and other rural villages when the Tuver project services were launched.

The University of Pittsburgh team (in partnership with community members, partners, and district health officials) conducted a needs assessment in September 2016, which identified access to healthcare and communicable and non-communicable diseases as significant problems. They also identified maternal and child health, adolescent health, skin diseases, and geriatric care as areas of concern. The village had very few toilets and limited street lighting and most homes lacked electricity, running water, and sufficient ventilation. Last, insufficient employment and income were highlighted as important issues for the local population. Important strengths included rich social networks within the community and village leadership committed to creating change for residents.

**Fig 1:**
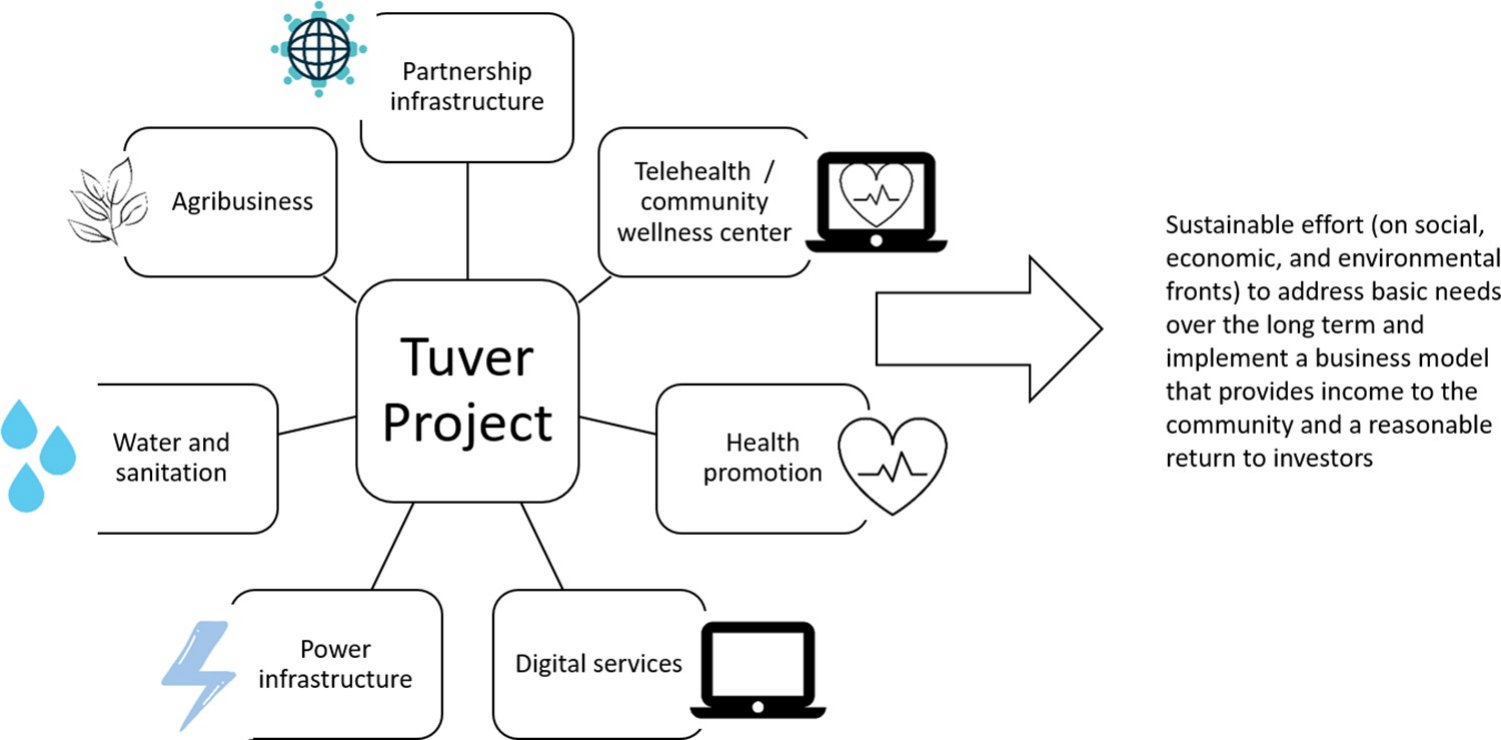
The seven components and goals of the Tuver project.

Given these needs and local resources, the project was designed to support sustainable, cross-sector change and increase health and well-being for residents of Tuver and surrounding villages, as summarized in Fig 1:

#### Component 1: Partnership structure

The partnership, which sought to be a coming together of equals, included:

- The University of Pittsburgh BoH team. Based at the business school, this team was responsible for developing the BoH approach [16]. The team also developed the Tuver project’s vision, secured funding, and oversaw project execution and evaluation.
- NLRDF (https://nlrdf.org). A registered non-governmental organization based in the western Indian state of Gujarat, it focuses on stimulating sustainable social development that decreases societal inequities. NLRDF was created by Arvind Limited and has decades of experience with rural community projects and rich relationships and credibility with state governments, local governments, and local nonprofits [23,24]. The organization served as a project advisor and a communication channel between the community and other partners and between the partnership and government agencies.
- Local leadership. Participants included the Tuver village’s leader (the Sarpanch or head of the Panchayat, an elected council consisting of 5 members) and the village accountant and record keeper (the Talati, who was appointed and employed by the state government). They joined the partnership to ensure that community needs were prioritized and to provide local credibility for the project.
- ATNF (www.atnf.org). An 80G and 12AA registered nonprofit organization, ATNF is a leader in telehealth in India. ATNF contributed expertise in designing and sustaining technology-enabled community healthcare programs to the Tuver Project. It led the project’s telehealth, pharmacy, and health education aspects and supported digital services through the Common Services Center. ATNF was responsible for human resources, technology solutions for telehealth, internet connectivity, infrastructure setup, training, quality assurance, community engagement, and monitoring and evaluation.
- SRS (https://safeworldrs.com/). This firm strives to enable inclusive and sustainable growth through innovative, environmentally friendly, and sustainable technology use. SRS was the design-build contractor for the project’s infrastructure, including the system that converts solar energy into Direct Current (DC) power, electrification of households, water, and civil work and structures. SRS also designed and executed the project’s agribusiness component.

#### Component 2: Telehealth services and community wellness center

The center housed an examination room with telehealth equipment for specialty consultations, a diagnostic laboratory, a pharmacy, and DC-powered refrigerators and air conditioners. The waiting room doubled as an education area.

#### Component 3: Health promotion

Community health workers delivered outreach to women and families about non-communicable disease prevention and treatment and women’s health. They also conducted health profiling to capture basic health history and establish baseline markers.

#### Component 4: Digital services

The Common Services Center supported Government of India e-services designed for rural and remote locations. Located in the wellness center, the center offered high-speed internet access with computers and printers and opportunities to pay utility bills, access banking and insurance services, engage with education and training opportunities, access health services, and utilize agriculture supports [25].

#### Component 5: Sustainable power

The project supplied locally generated, solar-powered electricity and compatible DC appliances, DC microgrids, and street lighting. In addition, it issued the poorest households 3 LED lights, a fan (for ventilating indoor smoke), charger outlets, and computerized meters to help users use energy efficiently.

#### Component 6: Water and sanitation

The initiative provided running water and community toilets with biodigesters built to European Union standards.

#### Component 7: Agribusiness

The project set up a suite of agricultural activities, such as sustainable crop-rotation farming, a nursery, a watershed, runoff collection, and a dairy operation using indigenous cattle that produced A2 milk, which commanded higher market prices than typical commercial milk.

### Study design

The design was informed by guidance for process evaluations of complex interventions and impact assessment for cross-sector partnerships, which typically include a detailed description of context, examination of implementation, evaluation of mechanisms, and short- and long-term outcomes. We focus here on context and partnership activities, given the early nature of the assessment [11,26]. The central goals for the analysis were to examine and explore the processes by which a BoH approach could support the development of a sustainable, cross-sector effort centered on telehealth, as well as the challenges of using such an approach. Accordingly, we emphasized 1) telehealth offerings, 2) partnership development and function, and 3) potential for the model’s long-term sustainability. To explore the potential for sustainability, we focused on core BoH concepts, including partnerships and the development of a sustainable and profitable business model [16]. We also drew on the conceptualization of sustainability in the implementation science literature as highlighted in the Exploration, Preparation, Implementation, Sustainment framework and Clinical Sustainability Assessment Tool, focusing on implementers and implementing organizations [27,28].

### Data collection and analysis

We gathered five main sources of data from the University of Pittsburgh team, ATNF, and SRS. Data related to telehealth services, Common Services Center offerings, and health promotion activities came from monthly service delivery reports and other documentation submitted by ATNF. Service provision data related to power infrastructure, water and sanitation, and the agribusiness venture came from reports from SRS. Additional data came from field notes from observational visits and minutes of meetings that included study team members. Data cover the planning period of the project until the COVID-19 pandemic created a shift in operations (September 2016 –April 2020). Although beyond the scope of this paper, we note that services were only paused in 2020 during the mandatory government lockdown, but otherwise shifted to accommodate external conditions.

We were guided by a case study approach, where the single, instrumental, case was defined as the Tuver Project and the phenomena of interest were the factors that impacted the sustainability of a BoH approach to deliver telehealth in a rural, Indian setting [29,30]. An implementation scientist (the lead author) was the primary analyst and analyzed the data in consultation with the ATNF, SRS, and University of Pittsburgh teams. We utilized analytic memos and matrices to support categorization and pattern-matching activities [29]. We also drew on complementary sources to support triangulation of data [31]. Data were managed using the Microsoft Office suite. The study team was composed of Indian nationals and Non-Resident Indians with extensive experience in health and healthcare among marginalized populations in India and with complementary perspectives from business, public health, engineering, and medical backgrounds. The team included individuals who spent months on-site to set up and support project activities. However, the analytic activities were also influenced by team members’ positions as privileged outsiders in the context of a rural, tribal village. We attempted to balance those etic perspectives with the composition of the partnership team, including local and village leadership. The Harvard University Area Institutional Review Board deemed the study Non-Human Subjects Research and thus the need for consent procedures was waived.

## Results

### Service outputs

#### Telehealth and wellness services

In the first 14 months, the program delivered a broad range of health-related services, as seen in Table 1.

**Table 1:** Distribution of services provided between February 2019 and April 2020.

	Number of services delivered	Mean per month (standard deviation)	Range per month
**Telehealth services**			
Specialty teleconsultations	1437	96 (35)	10–150
Laboratory tests	568	38 (14)	4–59
Pharmacy	979	65 (29)	0–105
**Health promotion**			
Health profiling	3928	262 (14)	4–59
Social health education	742	49 (45)	0–129

The program delivered clinical services to patients from newborns to seniors and conducted health profiling among adults. Social Health Education initially targeted adult women and was later expanded to girls in the secondary-level school age. As seen in Table 2, a total of 1437 teleconsultations were conducted, mainly by family physicians, dermatologists, and orthopedic surgeons.

**Table 2:** Distribution of teleconsultations provided between February 2019 and April 2020 (n = 1437).

Specialty	Total teleconsultations	Percent of total (%)
Family Physician	828	58
Dermatology	259	18
Orthopedics	148	10
Pediatrics	104	7
Gastroenterology	32	2
Cardiology	19	1
Obstetrics and Gynecology	17	1
Respiratory Medicine	11	1
Urology	9	1
Ophthalmology	8	1
Endocrinology	1	0
Neurosurgery	1	0

The Tuver Project served residents in a broad catchment area. For example, patients who received specialty and super specialty teleconsultations came from 94 villages. Table 3 offers a summary of the main villages served by the project’s health services. Several factors likely explain these villages’ higher utilization rates: Tuver is relatively accessible to them, they were the first to receive the project’s targeted outreach, and the head (Sarpanch) of each was sensitized to the project goals from the outset.

**Table 3:** Geographical distribution of key services.

Village	Percent of patients receiving specialty and super specialty consultations (%)	Percent of participants engaged in health profiling activities (%)	Percent of participants who received Social Health Education (%)
Tuver	32	12	17
Unchidhinal	15	14	21
Gadhda Shamlaji	7	17	15
Vikhran	7	0	0
Gundel	7	15	12
Kanthapura	0	9	8
Chada	0	8	8
Remaining villages	32	25	19

#### Non-health services

The Common Services Center served adults and performed 741 total transactions, averaging 49 per month (SD = 45) with a range of 16–175 transactions. The infrastructure developed included running water and toilets, as well as DC power generated by solar energy, which supported the delivery of digital services, community lighting, water and sanitation, and agribusiness. For example, locally generated power supported the agribusiness by powering submersible pumps drawing water for irrigation from tube wells.

### Influences on sustainability

In addition to services delivered, we identified four important potential drivers of sustainability for the Tuver Project.

#### Power-sharing and aligning partners’ goals and values offer promise for sustainability

While the partnership sought to act and be perceived as an alliance of equals, the fact that the University of Pittsburgh team as the funder/investor had control over the initial financial resources increased the potential for creating a Gramscian hegemony, in which the partner with the most resources/power sets the norms and goals for the effort [32]. Instead, the University of Pittsburgh team coordinated joint action based on the ideational power stemming from the alignment of goals and values among the partners. For example, community members, village administrators, and local government representatives reported improvements in villagers’ quality of life. NLRDF and SRS recognized the project’s alignment with their missions and an opportunity for experimentation with innovative solutions. ATNF saw the potential for adapting its telehealth solutions for markets lacking infrastructure and expanding rural offerings beyond the treatment of acute issues to incorporate prevention and wellness. For the University of Pittsburgh team, the Tuver project offered the opportunity to test the validity of the BoH strategy. At the same time, the reality that the University of Pittsburgh team provided the funding via separate contracts with NLRDF, ATNF, and SRS caused these partners to view the University of Pittsburgh team as the central communication node and leader of the work. They initiated consultations about changes and innovations with the University of Pittsburgh team’s leadership, which moderated discussions across the partnership. The exception to this communication pattern was village leadership, which communicated to the other partners through NLRDF due to the inability of other partners to speak local languages.

#### A synergistic business model supports the long-term delivery of services

The University of Pittsburgh team provided all capital expenditures for the wellness center’s construction, as well as telehealth equipment, personnel recruiting and training, power and water infrastructure, and operating expenses for the first two years. The model was designed to be self-sustaining after two years of operation by 1) reimbursing services through government programs such as health insurance and 2) returns from the agribusiness enterprise that provide income and employment to the local community and generate enough economic value to support the Tuver Project. At the end of Year 1, all healthcare services, including specialty and super-specialty teleconsultations and basic laboratory tests at the wellness center were available at no cost. The project provided medicines free of charge to those with incomes below the poverty line and at discounted rates to all other patients. The program offered outreach services and menstrual hygiene kits at no cost to community members. The Common Services Center provided services at nominal prices predetermined by the relevant governmental authority.

SRS designed the agribusiness enterprise to generate revenue from profitable cash crops to complement local food production and animal husbandry. Proceeds were earmarked for operating the wellness center and maintaining the power, water, and sanitation infrastructure. Current projections estimate a 17% return on investment, which is sufficient to meet sustainment goals. The agribusiness also provided participating families with lease payments for their land and wages if they chose to work for the agribusiness. Additionally, SRS trained other local farmers in advanced irrigation methods, organic fertilizing and pest control, and effective crop-rotation techniques to increase their farms’ economic and environmental sustainability.

#### Local hiring and ongoing monitoring and training supported high-quality implementation and potential for sustainability

The partners made a conscious decision to hire, train, and support local staff to improve implementation and sustainability. ATNF recruited and trained community members to serve as administrators for the Common Services Center and as paramedics. They also built weekly monitoring and supervision into their processes, leveraging data from electronic medical records, software platforms used to deliver services, and staff reports. This allowed them to ensure that high-fidelity delivery of telehealth, health promotion, and digital services. SRS used a similar model and recruited, trained, and oversaw local workers and supervisors for the infrastructure and agribusiness activities. They utilized regular monitoring of the performance of the power, household lighting and ventilation, streetlights, water, and sanitation infrastructure to ensure high-quality implementation. Both ATNF and SRS offered ongoing training and professional development for staff members.

#### Limited resonance of highly technical services with the community hindered implementation and poses a challenge to sustainability

Rural and tribal communities in and around Tuver were new to technology-based healthcare and digital services. Despite attempts to engage and prime potential users for these services, a series of barriers limited initial uptake. First, community members were unfamiliar with telehealth and preventive services. Second, local norms promoted engagement with historically trusted providers and healers. Third, low literacy rates in tribal and rural communities and multiple tribal languages made effective communication a challenge for the central team. Finally, cultural gaps between specialists from urban multi-specialty hospitals and patients from rural, tribal villages posed a challenge. The team attempted to address many of these challenges through ongoing interaction and outreach efforts. The team also relied on local staff members to serve as intermediaries and bridge potential divides.

Separately, a series of infrastructure-related challenges arose. For example, women were reluctant to use the toilets and toilet taps were sometimes left open, causing the tanks to run out of water. Similar inefficiencies emerged in the use of household electric power and water from SRS-dug tube wells that supply the toilets and potable water. The team addressed these challenges through responsive engagement with local leaders and community members.

## Discussion

This study examined early results from a Business of Humanity project designed to deliver telehealth services in a rural village in Gujarat, India and found potential for a systems-focused, partnership-based approach to addressing health and well-being. This connects with the move among significant segments of the corporate community towards emphasizing the importance of “purpose” as a goal that transcends profits and provides a foundation for sustained action [33–35]. At the same time, the results highlight challenges related to sustaining a complex intervention among diverse partners, including individuals from historically marginalized communities.

### Takeaway #1: Engagement of diverse, core partners through alignment of values and inspiration increases the likelihood of sustainability of telehealth and linked services

Each partner offered complementary, essential competencies, which gave them significant material and bargaining power. The prime partner (the University of Pittsburgh team) strategically employed ideational power, which emphasizes alignment of values and aspirations, to share power and ensure that the unique goals and needs of various partners were met. The use of ideational power is vital for cross-sector partnerships as it can attract new actors and resources, thereby amplifying successes and impact [36]. The findings related to power-sharing are consistent with conceptualizations of innovation-focused partnerships, which emphasize shared interests, a clearly articulated purpose, a common understanding of available resources, and a clear role through which each actor can demonstrate unique strengths [37]. At the same time, given the complexity of the partnership structure, unanticipated issues and challenges will inevitably arise and the intricate manner in which project components link will require monitoring and structured, ongoing communication between the partners to sustain the work. Another challenge relates to power differentials between partners, particularly in the context of partnerships with marginalized groups.

### Takeaway #2: The development of health-promoting ecosystems can support the use of telehealth services

Telehealth services were delivered as part of an effort that addressed the broader health-promoting context and generated economic value, employment, and income for community members. This attention to context and systems thinking offers the opportunity to improve access to care while addressing some of the social determinants of health that drive poor health outcomes for tribal, rural populations [38]. At the same time, the importance of the supporting system is echoed in the literature, which highlights the need to build a partnership base for financing and ensuring sustained delivery of telehealth services instead of relying on a vertical approach [39].

### Takeaway #3: Stakeholder engagement can be challenging for highly technical solutions, but local intermediaries can bridge disconnects to support engagement

Engaging community members and local leaders deeply proved challenging due to language differences, power dynamics, cultural disconnects, and the community’s unfamiliarity with telehealth and other digital services. These challenges are consistent with patient- and community-level barriers identified in the literature and also reflect the structural inequities that rural, tribal villages face [9,40]. For communities with deep ties to local, traditional healers, additional supports and customization may be required to make the service a useful complement and extension of existing care [38]. Locally hired staff bridged cultural and other divides between community members and service providers. Similarly, partnering with a trusted local foundation (NLRDF) allowed for vital relationships to be built, but as with any brokered relationship, added a layer of mediation between partners. Ensuring that the ultimate users of the service and relevant community leaders influence the project in a more directed manner will be critical for sustainability [41,42].

As with any study, the results must be placed in the context of limitations. First, these cross-sectional results are early and highlight opportunities to build sustainable change but do not evaluate that change. This is a particular challenge, given that “pilotitis” is an ongoing issue with digital health solutions in low- and middle-income countries [37]. Second, the data emphasize the perspectives of project leaders rather than community members and reflect an outsider perspective. This was mitigated in part by the emphasis on data from ATNF and SRS staff who lived near the village for the months preceding service launch and were on-site regularly. Planned data collection with community members and local leaders is expected to resume when appropriate in relation to COVID-19. Last, this process evaluation covers a single demonstration project and the findings may be idiosyncratic. At the same time, the study’s strengths outweigh these limitations. First, the study offers a novel approach for delivering telehealth services in rural India by linking with health promotion, digital services, agribusiness, power infrastructure, and water/sanitation. Such an approach attends to the concern that much of global development focuses narrowly on technical solutions without intervening on infrastructure and context [40]. Second, it relied upon multiple sources of data and complementary viewpoints to increase the credibility of the results and potential transferability of the model generated. Third, the study contributes to an identified gap in the literature: an examination of cross-sectoral partnerships in low- and middle-income countries [14].

The timing is right for further research into partnered, systems-focused approaches as shifts in broader policies and political forces, such as the Ayushman Bharat program to increase healthcare access, may provide a window to create change where one had not existed previously [43]. As an example, the Tuver Project’s goals are aligned with the national program’s focus on strengthening preventive and primary care services. Additionally, the BoH approach could offer a chance to leverage public-private partnerships that are part of the Ayushman Bharat program, but with a grounding in community engagement, oversight, and sustainable funding that protects equitable access to healthcare for the underserved [22].

Overall, the Tuver demonstration project highlights the potential for a Business of Humanity approach to bring diverse, complementary, committed partners together for coordinated, community-level action to improve the health and well-being of a marginalized community over the long term. A model such as this increases the likelihood that efforts will be sustainable but requires deep investments from a range of partners and stakeholders with aligned goals, visions, and values.

## References

[1] Jennett P, Hall LA, Hailey D, Ohinmaa A, Anderson C, Thomas R, et al. The socio-economic impact of telehealth: a systematic review. J Telemed Telecare. 2003;9(6):311–20. doi: 10.1258/135763303771005207 14680514

[2] World Health Organization. Telemedicine: opportunities and developments in member states. Report on the second global survey on eHealth: World Health Organization; 2010.

[3] Ganapathy K. Distribution of neurologists and neurosurgeons in India and its relevance to the adoption of telemedicine. Neurol India. 2015;63(2):142. doi: 10.4103/0028-3886.156274 25947977

[4] Gopalakrishnan S, Immanuel AB. Progress of health care in rural India: a critical review of National Rural Health Mission. International Journal of Community Medicine and Public Health. 2018;5(1):4.

[5] Balarajan Y, Selvaraj S, Subramanian S. Health care and equity in India. The Lancet. 2011;377(9764):505–15. doi: 10.1016/S0140-6736(10)61894-6 21227492 PMC3093249

[6] McLean S, Sheikh A, Cresswell K, Nurmatov U, Mukherjee M, Hemmi A, et al. The impact of telehealthcare on the quality and safety of care: a systematic overview. PloS one. 2013;8(8). doi: 10.1371/journal.pone.0071238 23977001 PMC3747134

[7] Solberg KE. Telemedicine set to grow in India over the next 5 years. The Lancet. 2008;371(9606):17–8. doi: 10.1016/S0140-6736(08)60052-5 18183655

[8] Government of India. 12th 5-Year Plan (2012–2017), Social Sectors, Volume III. New Delhi, India: SAGE Publications; 2013.

[9] Scott Kruse C, Karem P, Shifflett K, Vegi L, Ravi K, Brooks M. Evaluating barriers to adopting telemedicine worldwide: A systematic review. J Telemed Telecare. 2018;24(1):4–12. doi: 10.1177/1357633X16674087 29320966 PMC5768250

[10] Ministry of Health and Family Welfare—Government of India. Meeting people‟s health needs in rural areas, Framework for Implementation. 2005–2012. New Delhi, India: National Rural Health Mission,; 2012.

[11] Van Tulder R, Seitanidi MM, Crane A, Brammer S. Enhancing the impact of cross-sector partnerships. Journal of Business Ethics. 2016;135(1):1–17.

[12] Babiak KM. Criteria of effectiveness in multiple cross-sectoral interorganizational relationships. Eval Program Plann. 2009;32(1):1–12. doi: 10.1016/j.evalprogplan.2008.09.004 19026446

[13] Tanwar R, Ojha R, Agarwal M, Singh A, Joshi H. Role of public-private partnerships in delivering health Care Services in India. International Journal of Advanced and Integrated Medical Sciences. 2016;1(3):116–8.

[14] Glandon D, Meghani A, Jessani N, Qiu M, Bennett S. Identifying health policy and systems research priorities on multisectoral collaboration for health in low-income and middle-income countries. BMJ global health. 2018;3(Suppl 4). doi: 10.1136/bmjgh-2018-000970 30364329 PMC6195136

[15] Andrews R, Entwistle T. Does cross-sectoral partnership deliver? An empirical exploration of public service effectiveness, efficiency, and equity. Journal of public administration research and theory. 2010;20(3):679–701.

[16] Camillus J, Bidanda B, Mohan NC. The business of humanity: strategic management in the era of globalization, innovation, and shared value: CRC Press; 2017.

[17] Office of the Registrar General & Census Commissioner I. Primary Census Abstract Data Tables—Gujarat: Government of India; 2020 [updated May 5, 2020. Available from: http://censusindia.gov.in/pca/pcadata/Houselisting-housing-Gujarat.html.

[18] Narain JP. Health of tribal populations in India: How long can we afford to neglect? The Indian journal of medical research. 2019;149(3):313–6. doi: 10.4103/ijmr.IJMR_2079_18 31249192 PMC6607830

[19] Mohindra K, Labonté R. A systematic review of population health interventions and Scheduled Tribes in India. BMC public health. 2010;10(1):438. doi: 10.1186/1471-2458-10-438 20659344 PMC2919477

[20] Maity B. Comparing health outcomes across Scheduled Tribes and Castes in India. World Development. 2017;96:163–81.

[21] Ministry of Health and Family Welfare—Government of India. Rural Health Statistics—2019: State/UT-wise Number of SCs, PHCs & CHCs Functioning in Rural Areas- I during 2005 and 2019. Ministry of Health and Family Welfare: Department of Health and Family Welfare; 2019.

[22] Gopichandran V. Ayushman Bharat National Health Protection Scheme: an Ethical Analysis. Asian Bioethics Review. 2019;11(1):69–80. doi: 10.1007/s41649-019-00083-5 33717301 PMC7747303

[23] Tripathi D. Alliance for change: A slum upgrading experiment in Ahmedabad: Tata McGraw-Hill Publishing Company Limited; 1998.

[24] Tripathi D, Jumani J. Change after alliance: sequel to Alliance for Change: Tata Mcgraw-Hill Publishing Company; 2001.

[25] Government of India—Ministry of Electronics and Information Technology. Common Services Centers 2019 [Available from: https://meity.gov.in/content/common-services-centers-0.

[26] Moore GF, Audrey S, Barker M, Bond L, Bonell C, Hardeman W, et al. Process evaluation of complex interventions: Medical Research Council guidance. BMJ. 2015;350. doi: 10.1136/bmj.h1258 25791983 PMC4366184

[27] Luke DA, Calhoun A, Robichaux CB, Elliott MB, Moreland-Russell S. Peer reviewed: the program sustainability assessment tool: a new instrument for public health programs. Preventing Chronic Disease. 2014;11. 10.5888/pcd11.130184 PMC3900326 24456645

[28] Aarons GA, Green AE, Palinkas LA, Self-Brown S, Whitaker DJ, Lutzker JR, et al. Dynamic adaptation process to implement an evidence-based child maltreatment intervention. Implementation science: IS. 2012;7(1):32. 22512914 10.1186/1748-5908-7-32 PMC3436717

[29] Stake RE. The Art of Case Study Research. Thousand Oaks, CA: SAGE Publications; 1995.

[30] Baxter P, Jack S. Qualitative Case Study Methodology: Study Design and Implementation for Novice Researchers. The Qualitative Report. 2008;13(4):544–59.

[31] Flick U. Triangulation in qualitative research. In: Flick U, vonKardorff E, Steinke I, editors. A companion to qualitative research 2004. p. 178–83.

[32] Menga F. Power and water in Central Asia: Routledge; 2017.

[33] Fink L. Letter to CEOs: Profit & Purpose. New York, NY: BlackRock; 2019.

[34] Updated Statement on the Purpose of a Corporation [press release]. Washington, DC, August 19 2019.

[35] Yunus M. Creating a world without poverty: Social business and the future of capitalism. Global Urban Development Magazine. 2009;4(2):16–41.

[36] Nye JS Jr. Public diplomacy and soft power. The annals of the American academy of political and social science. 2008;616(1):94–109.

[37] National Academies of Sciences E, Medicine. Value Proposition and Innovative Models for Multi-Sectoral Engagement in Global Health: Proceedings of a Workshop: National Academies Press; 2020. 32352688

[38] Gurney J, Fraser L, Ikihele A, Manderson J, Scott N, Robson B. Telehealth as a tool for equity: pros, cons and recommendations. The New Zealand Medical Journal (Online). 2021;134(1530):111–5. 33651781

[39] Macabasag RLA, Magtubo KMP, Marcelo PGF. Implementation of telemedicine services in lower-middle income countries: lessons for the Philippines. Journal of the International Society for Telemedicine and eHealth. 2016;4:e24 (1–11).

[40] Snell-Rood C, Jaramillo ET, Hamilton AB, Raskin SE, Nicosia FM, Willging C. Advancing health equity through a theoretically critical implementation science. Translational Behavioral Medicine. 2021. doi: 10.1093/tbm/ibab008 33904908 PMC8367016

[41] Trickett EJ. Multilevel community-based culturally situated interventions and community impact: An ecological perspective. Am J Community Psychol. 2009;43(3–4):257–66. doi: 10.1007/s10464-009-9227-y 19333751

[42] Ramanadhan S, Davis MM, Armstrong RA, Baquero B, Ko LK, Leng JC, et al. Participatory implementation science to increase the impact of evidence-based cancer prevention and control. Cancer Causes Control. 2018;29(3):363–9. doi: 10.1007/s10552-018-1008-1 29417296 PMC5858707

[43] Labrique AB, Wadhwani C, Williams KA, Lamptey P, Hesp C, Luk R, et al. Best practices in scaling digital health in low and middle income countries. Global Health. 2018;14(1):103. doi: 10.1186/s12992-018-0424-z 30390686 PMC6215624

